# Prolonged grief disorder in Arabic-speaking treatment-seeking populations: Relationship with socio-demographic aspects, loss- and trauma-related characteristics, and mental health support

**DOI:** 10.3389/fpsyt.2022.933848

**Published:** 2022-09-15

**Authors:** Freya Specht, Max Vöhringer, Christine Knaevelsrud, Birgit Wagner, Nadine Stammel, Maria Böttche

**Affiliations:** ^1^Research Department, Center ÜBERLEBEN, Berlin, Germany; ^2^Clinical Psychological Intervention, Freie Universität Berlin, Berlin, Germany; ^3^Clinical Psychology and Psychotherapy, Medical School Berlin, Berlin, Germany

**Keywords:** prolonged grief, Arabic-speaking populations, mental health support, ICD-11, associated factors

## Abstract

**Introduction:**

Prolonged grief disorder (PGD) has been included as a new diagnosis in the ICD-11 and is set to be included in the DSM-5-TR. To better identify vulnerable individuals, different factors associated with PGD have to be taken into account, but results regarding these factors remain equivocal. Moreover, few studies on PGD are available from Arabic-speaking populations and from different countries dealing with conflicts and wars. The objective was thus to examine PGD prevalence and associated characteristics in these populations.

**Materials and methods:**

A total of *N* = 1,051 bereaved participants from Arabic-speaking populations completed the PG-13 as part of a screening procedure for an online mental health intervention. Multiple linear regression was conducted to examine associated factors for PGD symptom severity, and multiple logistic regression was applied to investigate associated factors for PGD according to PG-13 diagnostic criteria.

**Results:**

Of the participants, 18.8% (*n* = 198) met the PGD diagnostic criteria, at an average of about 6 years post-loss. The multiple linear regression yielded eight associated factors for PGD symptom severity (age, gender, number of losses, number of traumatic event types, relationship with the deceased, age at loss, impairment during first year post-loss, perceived social support), which explained 40.2% of the variance [*F*_(17, N=1,033)_ = 40.82, *p* < 0.001, *R*^2^ = 0.402]. The multiple logistic regression yielded five significant associations with PGD (gender, relationship with the deceased, number of lost persons, impairment during first year post-loss, perceived social support), which explained 33.0% (Nagelkerke *R*^2^) of the variance in PGD according to PG-13 diagnostic criteria.

**Discussion:**

A substantial proportion of the participants met the PG-13 criteria for PGD, emphasizing that therapeutic services are indispensable in this population. The associated factors for PGD found in our Arab-speaking sample are largely consistent with those found in studies from other regions. The slightly differing numbers of associated factors between the linear and logistic regression underline that a continuous score reflects the continuum between normal and dysfunctional grieving, and therefore also a range of factors associated with PGD.

## Introduction

Recent research has shown that a significant proportion of bereaved people experience a severe and stressful grieving process, in which the intensity of grief-related distress does not decrease over time (1, 2). Due to these distressing and debilitating symptoms, Prolonged Grief Disorder (PGD) has been included in the International Classification of Diseases [ICD-11; (3)] as a distinct mental disorder in the section of disorders specifically associated with stress. Here, the disorder is defined as a persistent and profound grief response characterized by longing or persistent preoccupation with the deceased. This is accompanied by intense emotional pain and the grief response lasts for an atypically long period of time after the loss (more than 6 months) and exceeds expected social, cultural, or religious norms [ICD-11, (3)]. The disorder will also be included in the forthcoming text revision of the Diagnostic and Statistical Manual of Mental Disorders [DSM-5-TR; (4)].

Meta-analyses have demonstrated that on average, about 10% of bereaved individuals develop a PGD after a natural loss (non-violent death causes, excluding suicides, murder, natural disasters, or terrorist attacks) of a close person (1), whereas nearly 50% develop a PGD after an unnatural loss [sudden and violent deaths, caused by accidents, suicides, homicides, disasters, terror, and war; (2)]. As a relatively new disorder, PGD has been investigated in many countries to date. However, the majority of these studies were conducted in Western and Eastern societies, and relatively few studies have focused on Arabic-speaking populations. As some Arabic-speaking regions are currently struggling with war and conflict and faced with a lack of mental health resources, it seems important to gather more knowledge on PGD in this population. In one of the few studies on PGD in Arabic-speaking countries, Al-Gamal et al. (5) investigated the prevalence of the disorder in a sample of 226 bereaved undergraduate students in Saudi Arabia. Over half of the participants indicated having lost a grandparent, and most losses were caused by illness. The authors found that 12% of the sample met the criteria for PGD based on a cut-off score of 32 on the Prolonged Grief Scale (PG-13). In another study with female students from the United Arab Emirates (UAE), 7.6% of the rather young sample met the PGD criteria (6) and in a sample of Syrian refugees residing in a Jordanian refugee camp, the PGD rate was 15.1% (7). In all three of these studies, deaths were mainly due to natural causes.

To better identify individuals who may be vulnerable to PGD, socio-demographic and loss- and trauma-related variables, as well as support structures, need to be taken into account. However, the results regarding these risk factors associated with PGD are still equivocal and there are only a small number of robust factors for which evidence has been repeatedly reported.

Regarding socio-demographic characteristics, there is some evidence that women are at greater risk of developing PGD after loss compared to men. For instance, in meta-analyses on the consequences of unnatural as well as violent loss, Djelantik et al. (2) and Heeke et al. (8) reported that female gender was associated with increased levels of PGD. Furthermore, some studies found that the age of the bereaved person seems to be a relevant risk factor, with younger age being associated with higher PGD symptoms (9–11). However, the meta-analyses by Djelantik et al. (2) found no significant associations with age, while Lundorff et al. (1) found a trend with older age being associated with higher prevalence of PGD and Heeke et al. (8) reported that older age is associated with more pronounced PGD symptoms only in persons who experienced an individual as opposed to a collective loss. In addition to younger age, in their review, Burke and Neimeyer (11) found evidence that having less education, little income, and being non-Caucasian predicted prolonged and intense grief. They also identified intrapersonal predictors such as avoidant, anxious, and insecure attachment styles as well as high levels of neuroticism.

Loss- and trauma-related aspects have been shown to be reliable risk factors for PGD across various studies. For instance, sudden and unexpected as well as violent losses (i.e., type of loss) such as accidents or suicides were associated with an increased risk of developing PGD (11–15). Additionally, some studies identified the experience of prior losses or multiple losses (i.e., number of losses) as further risk factors for PGD (8, 11, 14). Bereaved persons who have experienced a higher number of traumatic events have been found to generally show increased levels of PGD (8, 14). This association may be particularly relevant for war-torn societies, where experiences of trauma and loss often co-occur. Also, discovering the body in cases of violent death or dissatisfaction with the death notification were identified as risk factors (11). In terms of the relationship with the deceased, several studies have revealed that the loss of close family members such as children, siblings, parents, or spouses, and high levels of pre-death marital dependency is associated with more severe PGD compared to the loss of distant relatives (8, 11). Results regarding the association between time since loss and the severity of PGD symptoms are inconsistent. In the meta-analysis by Heeke et al. (8) on violent losses, this association was only established as a trend. By contrast, Djelantik et al. (2) found that a shorter time since an unnatural loss was associated with more pronounced PGD symptomatology, and Lundorff et al. (1) found no association between the length of elapsed time after a loss and PGD. Moreover, functional impairment seems to play an important role when investigating PGD. For instance, Nielsen et al. (16) found that higher scores on a questionnaire assessing self-perceived functional impairment were associated with higher symptoms of PGD in bereaved persons.

In addition to socio-demographic and loss-related variables, a lack of support structures may yield another association with PGD. Receiving social and/or professional support improves ones individual's wellbeing, and in particular, support from family members and friends seems to be essential after experiencing a loss. However, only a relatively small number of recent studies have investigated the relationship between support and bereavement. Burke and Neimeyer (11) found evidence for low levels of social support predicting intense grieving. Lower perceived social support after suicide loss was also associated with increased levels of PGD (17), whereas a longitudinal study from Norway investigating bereaved parents after a terror attack found no such association (18). Another study also reported a negative association in a sample of students in Saudi Arabia (5). The efficacy of grief-related psychological interventions has already been demonstrated (19). However, the availability of professional support certainly varies across different countries (20).

In summary, PGD prevalence rates and risk factors differ across studies. This discrepancy and lack of clarity may be attributable to different samples (e.g., country of origin and cause of death), designs (e.g., measurements and evaluation) and contextual factors of the individual studies. One general aim of the new ICD-11 classification is to improve the clinical utility and global applicability, also focusing on low- and middle-income countries (21). Although some international studies on PGD have already been conducted, very few studies on PGD are available from Arabic-speaking populations. In these populations, however, research on PGD seems to be particularly essential due to the long-standing unstable political situation, in which both natural and unnatural losses are experienced. Another aspect regarding the potential differences in risk factors reported across the research seems to be the evaluation method applied. When investigating risk factors for PGD, it is recommended that researchers assess not only PGD symptom severity (by using continuous PGD symptom scores) but also apply PGD diagnostic criteria. This approach should yield more clinically relevant findings, as it focuses on risk factors for clinical samples rather than investigating a broader range of psychological reactions to bereavement (10). Indeed, Singer et al. (10) revealed that these two analytical strategies may lead to different results.

The objective of the current cross-sectional study was therefore to examine PGD prevalence and associated factors of PGD with respect to three different aspects [(1) socio-demographic variables, (2) loss- and trauma-related variables, (3) mental health support] in a sample of bereaved individuals from Arabic-speaking populations by analyzing both PGD symptom severity and the PG-13 diagnostic criteria which closely resemble ICD-11 PGD. We sought both to underpin existing findings and to provide initial results on the cross-national prevalence and associated factors from Arabic-speaking populations. Besides focusing on associated factors *per se*, an additional goal of the study was to compare the results based on the application of the two different analytical approaches (continuous vs. categorical outcome). Because of the cross-sectional design of the study, the results refer to associated factors of PGD rather than risk factors.

## Materials and methods

### Sample and procedure

Participants were treatment-seeking individuals who completed an online screening survey to take part in an internet-based treatment for either post-traumatic stress disorder (PTSD) or depression in the Arabic language (approved by the Ethics Committee of the University of Leipzig, Germany: 236-11-22082011). The study has a cross-sectional design and is part of an open-label dissemination study of the included treatments [e.g., (22–24)]. The dissemination study is being implemented in the context of a program that has been ongoing since 2008 and is conducted by a German psychosocial center that offers psychosocial treatment for victims of torture and war. Participants were recruited through internet advertisements, e.g., social media and a program website. The program website provides general information about PTSD, depression, the treatments and alternatives, and the purpose of the study. Participants were informed about the voluntary nature of participation and that their data would be protected by rigorous security measures. No financial compensation was offered.

To be included in the present analysis, participants had to be able to read and write in Arabic, be aged 18 years or older and have access to the internet. Furthermore, they had to have experienced one or more losses of family members or friends. As only screening data were analyzed, the inclusion and exclusion criteria for the dissemination study of the treatments were not yet applicable [for exclusion criteria of the intervention see (22)].

Participants were recruited between September 2021 and March 2022. During this time, a total of 2,585 individuals registered, provided informed consent and completed the screening questionnaires about loss and grief. Of these, 1,234 participants (47.7%) reported having experienced a loss and thus qualified for the study. A total of 134 (10.9%) participants were excluded from the analysis because they indicated an inconclusive category for the cause of the loss they experienced, 16 (1.3%) did not complete the PG-13, and 33 (2.7%) reported unrealistic time periods regarding the question of how long ago they experienced the loss (i.e., dates preceding their date of birth). The final sample thus comprised *N* = 1,051 individuals. For the analytical approach using categorical outcomes, 94 of these participants (8.9%) had to be excluded from the sample as their loss was <6 months ago and they could thus not meet the duration criterion of PGD caseness according to PG-13 diagnostic criteria even if symptoms were severe.

### Measures

The screening questionnaire asked for socio-demographic data, i.e., age, gender, education, country of origin, country of residence, and included a binary question about whether participants had to flee from their home country as a consequence of an armed conflict or fear of persecution.

The Prolonged Grief-13 [PG-13; (25)] served as the basis for the two dependent variables measuring prolonged grief disorder: once as a continuous variable and once a categorical variable. The PG-13 contains 13 items, of which two are dichotomous (*yes*/*no*) and inquire about whether the respondent has experienced a high frequency (at least daily) and long duration (minimum 6 months) of primary symptoms and functional impairment. The remaining 11 items assess cognitive, behavioral and emotional symptoms and are rated on 5-point Likert scales ranging from 1 (*not at all*) to 5 (*several times a day*/*overwhelming*). The continuous measure of the PG-13 is obtained by summing up the 11 Likert-scaled items. This sum score has proven to be reliable and valid [e.g., (26, 27)]. In the present sample, the internal consistency of the Likert-scaled items was α = 0.93.

The categorical measure of the PG-13 assesses the presence of PGD according to the criteria of Prigerson et al. (25) which most closely resembles ICD-11 PGD. These include (A) the experience of a loss (inclusion criterion for this study), (B) separation distress: loss-related yearning and emotional pain at least daily (score of 4 or 5 on items 1 or 2), (C) at least 6 months duration of elevated symptoms of separation distress (answer *yes* on frequency and duration item), (D) at least five cognitive, behavioral and emotional symptoms at least daily (score of 4 or 5 on at least five of the Likert-scaled items), and (E) significant impairment in social, occupational, or other important areas of functioning (answer *yes* on impairment item).

Loss-related variables were assessed using single-item questions developed for the purpose of the present study, which asked about the loss and grief experience and whether and how many close persons had been lost. The following six items were then applied, which referred to the loss event that participants experienced as the most impairing: (1) Cause of death. Participants were asked about the cause of death with predefined response options: sudden natural death, prolonged illness, accident, suicide, violent crime, and abortion. Of these, suicide, violent crime and accident were considered as violent causes of death while the remaining options were considered as non-violent causes of death. (2) Relationship with the deceased: other relatives, good friend, grandparent, or close family, with the latter defined as parent, sibling, spouse, or child. (3) Time since loss. (4) Age at loss. (5) Age of deceased. (6) Retrospective assessment of impairment of daily life during the first year after the loss, measured on a 5-point Likert scale ranging from 0 (*not at all*) to 4 (*very much*).

*Exposure to traumatic events* was indexed using a measure derived by combining the list of traumatic events from the Harvard Trauma Questionnaire [HTQ; (28)] and the Post-traumatic Diagnostic Scale [PDS; (29)]. In addition, we included item 16 (“Severe injury, damage or death inflicted on others”) from the Life Events Checklist for DSM-5 [LEC-5; (30)]. Altogether, the resulting measure comprised 24 items indexing exposure to various types of traumatic events. The sum score, representing the number of traumatic event types experienced by participants, was used in the analysis.

To assess mental health support, the following three measures were applied: (1) A single-item question (yes/no) asking whether participants had ever received therapeutic treatment because of their current problem for which they registered for the program. (2) The Multidimensional Scale of Perceived Social Support [MSPSS; (31)], which measures *perceived social support* by family, friends, and significant others with 12 items. Items are rated on a 7-point Likert scale ranging from 1 (*very strongly disagree*) to 7 (*very strongly agree*), with higher scores indicating greater perceived social support. The mean value of the 12 items constitutes the overall MSPSS score. The Arabic translation of the MSPSS used in the present study has proven to be reliable and valid (32) and the internal consistency in the present sample was α = 0.91. (3) To measure professional support structures on a country level, we approximated the *mental health workforce capacity* by calculating the mean number of psychiatrists and psychologists per 100,000 inhabitants in each country of residence. For this purpose, we summarized the latest available data from the World Health Organization (33, 34) and the Organization for Economic Cooperation and Development (35). Only for the country of Libya were no such data available in the WHO and OECD repositories, and we therefore used the figures provided by Okasha et al. (36). A summarized list of mental health workforce capacity for all countries of residence in the sample is provided in the Supplementary material.

### Statistical analyses

Only complete screening measures were included in the analyses; therefore, no missing data had to be excluded nor imputed. The analysis plan was two-fold: First, multiple linear regression was conducted to examine associated factors for PGD symptom severity. Second, multiple logistic regression was applied to investigate associated factors for PGD according to PG-13 diagnostic criteria. For the latter, all participants whose most impairing loss was <6 months ago were removed (*n* = 94), as they did not meet the duration criterion of PGD caseness. To include the categorical variables as predictors in both regression analyses, dummy variables were created. For the variable “relationship with the deceased,” “other relative” was selected as the reference category. Statistical analyses were conducted using the program R (version 3.6.2).

## Results

### Socio-demographic characteristics

A summary of all socio-demographic and loss-, trauma- and support-related characteristics of the sample is provided in Table 1. In the following, some of the results from Table 1 will be described in more detail. The sample consisted of *N* = 1,051 participants, of whom 773 (73.5%) were female. Participants were on average 25 years old (range 18–63 years). About 40% (*n* = 422) of the participants reported having a college diploma. The study included participants from 31 different countries of origin, with the five most frequent being Egypt (32.4%), Saudi Arabia (19.3%), Jordan (7.1%), Syria (6.2%), and Morocco (5.8%). Regarding the country of current residence, 45 different countries were reported, with the five most frequent being Egypt (32.3%), Saudi Arabia (18.5%), Jordan (7.4%), Morocco (5.6%), and Germany (3.9%) (see Supplementary material for a complete list of countries). A total of 8.0% of the participants indicated that they had to flee from their home country as a consequence of an armed conflict or fear of persecution.

**Table 1:** Demographic, loss-, trauma-, and support-related variables of participants.

**Variables**	**%, *M***	***SD***	**Range**
Age (*M*)	25.4	7.52	18–63
Gender: female (%)	73.5	-	-
Education: lower (%)	59.8	-	-
Migration status: flight experience (%)	8.0	-	-
Exposure to traumatic events (*M*)	4.0	3.91	0–23
Cause of death: violent (%)	17.6	-	-
Relation to lost person (%)
Other relatives	16.7	-	-
Good friend	11.5	-	-
Grandparent	34.6	-	-
Close family	37.2	-	-
No. of lost persons (*M*)	2.1	1.85	1–22
Time since loss (in months; *M*)	73.5	70.40	0–420
Age at loss (*M*)	19.5	8.54	0–80
Age of lost person (*M*)	53.3	23.00	0–120
Impairment during first year (*M*)	2.1	1.34	0–4
Perceived social support (MSPSS; *M*)	3.4	1.49	1–7
Previous treatment: received (%)	18.7	-	-
Mental health workforce capacity (*M*)^a^	3.9	11.08	0.009–112.275

*N* = 1,051.

MSPSS, Multidimensional Scale of Perceived Social Support.

a Mean number of psychiatrists and psychologists per 100.000 inhabitants per country of residence.

### Loss- and trauma-related characteristics

When asked which loss was most impairing, 37.2% (*n* = 391) of respondents reported the loss of a close family member, 34.6% (*n* = 364) the loss of a grandparent, 11.5% (*n* = 121) the loss of a good friend, and 16.7% (*n* = 175) the loss of another relative. Losing a parent, sibling, spouse, or child were subsumed under “close family members” (with *n* = 306, *n* = 66, *n* = 12, and *n* = 7, respectively). The average age of the deceased was 53.3 years.

Most causes of death were non-violent in nature (*n* = 866, 82.4%); of these, 53.0% (*n* = 557) were sudden natural deaths, 28.8% (*n* = 303) were due to prolonged illness, and 0.6% (*n* = 6) due to abortion. From the violent causes of death (*n* = 185, 17.6%), 1.1% (*n* = 12) were due to suicides, 2.9% (*n* = 30) were due to violent crimes, and 13.6% (*n* = 143) were due to accidents.

The mean sum score for PGD symptom severity on the PG-13 was *M* = 31.3, at an average of 6.1 years (73.5 months) post-loss.

In addition to the loss, participants reported that they had experienced, on average, four out of 24 different traumatic event types (range 0–23). The three most frequently reported traumatic event types were sexual contact while under the age of 18 with a person at least 5 years older (e.g., contact with genitals or breasts; *n* = 428, 16.3%), being close to death (*n* = 389, 14.8%), and poor health without access to medical care (*n* = 327, 12.5%; see Supplementary material for a detailed list of the traumatic event types).

### Mental health support

Only 18.7% (*n* = 197) of the participants had ever received any therapeutic treatment because of the symptoms that caused them to seek help by the program. Perceived social support, as measured with the MSPSS, lay on average at 3.4 (range 1–7). The mental health workforce capacity was on average 3.9 (mean number of psychiatrists and psychologists per 100,000 inhabitants per country of residence) and showed a very large range (0.009–112.275).

### Associated factors for PGD symptom severity

The multiple linear regression yielded eight factors associated with PGD symptom severity. As shown in Table 2, age, gender, number of losses and traumatic event types, relationship with the deceased, age at loss, impairment during first year, and perceived social support were all significantly associated with prolonged grief symptom severity. The model explained a total of 40.2% of the variance [*F*_(17, N=1,033)_ = 40.82, *p* < 0.001, *R*^2^ = 0.402, *R*^2^*Adjusted* = 0.392].

**Table 2:** Results of the multiple regression of demographic, loss-, trauma-, and support-related variables on the severity of prolonged grief.

**Variables**	***B***	***SE***	**β*^*a*^***	***p-*value**
Age	−0.26^**^	0.08	−0.16^**^	0.002
Gender: male (vs. female)	−3.60^***^	0.71	−0.29^***^	<0.001
Education: higher (vs. lower)	0.44	0.68	0.04	0.52
Migration status: flight experience (vs. no flight experience)	−0.52	1.21	−0.04	0.67
Exposure to traumatic events	0.30^***^	0.08	0.10^***^	<0.001
Cause of death: violent (vs. non-violent)	−0.06	0.93	−0.005	0.95
Relation with deceased (vs. other relatives)
Good friend	2.22	1.19	0.18	0.06
Grandparent	−0.62	1.03	−0.05	0.55
Close family	4.54^***^	0.93	0.37^***^	<0.001
No. of lost persons	0.55^**^	0.17	0.08^**^	0.001
Time since loss (in months)	−0.01	0.01	−0.05	0.22
Age at loss	0.15^*^	0.07	0.11^*^	0.03
Age of lost person	−0.003	0.02	−0.01	0.89
Impairment during first year	4.20^***^	0.23	0.46^***^	<0.001
Perceived social support (MSPSS)	−1.39^***^	0.20	−0.17^***^	<0.001
Previous treatment: received (vs. not received)	−0.41	0.78	−0.03	0.60
Mental health workforce capacity	0.01	0.03	0.01	0.60

*N* = 1,051.

MSPSS, Multidimensional Scale of Perceived Social Support.

* *p* < 0.05.

** *p* < 0.01.

*** *p* < 0.001.

a Dummy-coded independent variables were not standardized.

Regarding the socio-demographic variables, younger age (*p* < 0.01) and identifying as female (*p* < 0.001) were associated with PGD symptom severity. The other socio-demographic variables (education, *p* = 0.52; flight experience, *p* = 0.67) were not significantly related to PGD symptom severity.

In terms of the loss- and trauma-related variables, a higher number of losses (*p* < 0.01), older age at loss (*p* < 0.05), having lost a close as opposed to distant family member (*p* < 0.001), higher subjective retrospective impairment in daily life during the first year after the loss (*p* < 0.001), and a higher number of experienced or witnessed traumatic event types (*p* < 0.001) were related to PGD symptom severity. All other loss-related variables (having lost a good friend, *p* = 0.06, or grandparent, *p* = 0.55, both as opposed to distant relatives; time since loss, *p* = 0.22; age of deceased, *p* = 0.89) were not significantly associated with PGD symptom severity.

With respect to mental health support, less perceived social support (*p* < 0.001) was related to PGD symptom severity. The other support-related variables (having received previous treatment, *p* = 0.60; mental health workforce capacity, *p* = 0.60) did not yield significant associations with PGD symptom severity.

### Factors associated with PGD according to PG-13

From the entire sample, *n* = 198 (18.8%) participants met the threshold for PGD. Table 3 shows the demographic as well as loss-, trauma-, and support-related characteristics of individuals who met PGD criteria compared to individuals who did not meet these criteria.

**Table 3:** Demographic, loss-, trauma-, and support-related variables of participants with and without PGD according to ICD-11.

**Variable**	**No PGD**	**PGD**
	***n* = 759**	***n* = 198**
Age, *M* (*SD*)	25.42 (7.61)	25.69 (7.60)
**Gender**, ***n*** **(%)**
Female	536 (76.7)	163 (23.3)
Male	223 (86.4)	35 (13.6)
**Education**, ***n*** **(%)**
Lower	454 (79.8)	115 (20.2)
Higher	305 (78.6)	83 (21.4)
**Migration status**, ***n*** **(%)**
Flight experience	66 (81.5)	15 (18.5)
No flight experience	693 (79.1)	183 (20.9)
Exposure to traumatic events, *M* (*SD*)	3.82 (3.88)	4.64 (3.91)
**Cause of death**, ***n*** **(%)**
Non-violent	618 (78.3)	171 (21.7)
Violent	141 (83.9)	27 (16.1)
**Relation with deceased**, ***n*** **(%)**
Other relatives	134 (86.5)	21 (13.5)
Good friend	92 (86.0)	15 (14.0)
Grandparent	293 (88.0)	40 (12.0)
Close family	240 (66.3)	122 (33.7)
No. of lost persons, *M* (*SD*)	2.04 (1.82)	2.29 (1.94)
Time since loss (in months), *M* (*SD*)	83.57 (70.63)	68.47 (66.21)
Age at loss, *M* (*SD*)	18.86 (8.30)	20.31 (8.75)
Age of lost person, *M* (*SD*)	53.42 (23.14)	51.71 (21.85)
Impairment during first year, *M* (*SD*)	1.81 (1.26)	3.09 (1.16)
Perceived social support (MSPSS), *M* (*SD*)	3.51 (1.47)	3.01 (1.47)
**Previous treatment**, ***n*** **(%)**
Received	147 (79.9)	37 (20.1)
Not received	612 (79.2)	161 (20.8)
Mental health workforce capacity, *M* (*SD*)^a^	3.70 (9.67)	5.23 (16.14)

*N* = 957.

MSPSS, Multidimensional Scale of Perceived Social Support.

a Mean number of psychiatrists and psychologists per 100.000 inhabitants per country of residence.

The multiple logistic regression yielded five factors associated with PGD. As shown in Table 4, identifying as female, loss of a close family member, number of lost persons, perceived impairment of daily life during the first year after the loss, and perceived social support were all significantly related to PGD. The logistic regression model was statistically significant, $\chi_{\left(17,\text{N}=1,051\right)}^{2}$ = 226.50, *p* < 0.001 and explained 33.0% (Nagelkerke *R*^2^) of the variance in PGD.

**Table 4:** Results of the logistic regression of demographic, loss-, trauma-, and support-related variables on PGD according to ICD-11.

**Variables**	***B***	***SE***	**95%** ***CI*** **for odds ratio**		
			**Lower**	**Odds ratio**	**Upper**
Age	−0.02	0.03	0.93	0.98	1.04
Gender: male (vs. female)	−0.56^*^	0.24	0.35	0.57	0.91
Education: higher (vs. lower)	0.12	0.21	0.74	1.13	1.71
Migration status: flight experience (vs. no flight experience)	−0.27	0.39	0.34	0.76	1.60
Exposure to traumatic events	0.05	0.03	1.00	1.05	1.10
Cause of death: violent (vs. non-violent)	−0.18	0.29	0.46	0.83	1.46
Relation to deceased (vs. other relatives)
Good friend	−0.10	0.42	0.40	0.91	2.04
Grandparent	−0.35	0.35	0.36	0.71	1.40
Close family	1.04^***^	0.30	1.60	2.83	5.15
No. of lost persons	0.09^*^	0.05	1.00	1.10	1.20
Time since loss (in months)	−0.004	0.002	0.99	1.00	1.00
Age at loss	0.003	0.02	0.95	1.00	1.05
Age of lost person	0.01	0.01	1.00	1.01	1.02
Impairment during first year	0.73^***^	0.08	1.77	2.07	2.44
Perceived social support (MSPSS)	−0.22^***^	0.06	0.71	0.80	0.91
Previous treatment: received (vs. not received)	−0.18	0.25	0.51	0.84	1.35
Mental health workforce capacity	0.01	0.01	0.99	1.01	1.03

*N* = 957.

MSPSS, Multidimensional Scale of Perceived Social Support.

* *p* < 0.05.

*** *p* < 0.001.

With regard to the socio-demographic variables, the odds of persons identifying as male for meeting PG-13 diagnostic criteria for PGD were almost half the odds of female participants [OR = 0.57, 95% CI (0.35, 0.91)]. The other socio-demographic variables (age, education, flight experience) were not associated with PG-13 diagnostic criteria for PGD.

With regard to the loss- and trauma-related variables, the odds of individuals that lost a member of the close family were almost three times the odds of participants who lost a member of the distant family for meeting PG-13 diagnostic criteria for PGD [OR = 2.83, 95% CI (1.60, 5.15)]. Moreover, persons who had experienced a higher number of losses showed a greater likelihood of meeting the diagnostic criteria [OR = 1.10, 95% CI (1.00, 1.20)]. Lastly, a higher perception of impairment of daily life functioning in the first year after the loss was associated with a greater likelihood of meeting the diagnostic criteria for PGD [OR = 2.07, 95% CI (1.77, 2.44)]. All other loss- and trauma-related variables (exposure to traumatic events, cause of death, having lost a good friend or grandparent as opposed to distant relatives, time since loss, age at loss, age of deceased) were not associated with meeting PGD diagnostic criteria.

Concerning mental health support, persons who reported more perceived social support were less likely to meet the PG-13 diagnostic criteria for PGD [OR = 0.80, 95% CI (0.71, 0.91)]. The other support-related variables (having received previous treatment, mental health workforce capacity) were not associated with meeting PGD diagnostic criteria.

## Discussion

The present study examined the prevalence of PGD and its associated factors in a sample of bereaved treatment-seeking adults in Arabic-speaking populations. By conducting both a linear and a logistic regression analysis, we investigated associated factors with PGD symptom severity and with PGD according to PG-13 diagnostic criteria. The sample of the current study was rather young and well educated, and the majority were female. Most of the losses were non-violent and occurred within the close family (most participants specified a parent) or involved a grandparent.

### PGD prevalence

A total of 18.8% (*n* = 198) of participants met the PGD diagnostic criteria according to the PG-13, at an average of about 6 years post-loss. This prevalence is higher compared to previous studies, which reported a pooled prevalence of PGD of 9.8% in diverse bereaved populations who experienced non-violent loss [meta-analysis by (1)]. Indeed, Lundorff et al. (1) even showed that the PG-13 used in our study was associated with lower PGD rates (3.2%) compared to other diagnostic tools. Moreover, the prevalence in the present sample is also higher than that reported in other Arabic-speaking samples [15.1% in refugees in a camp in Jordan, (7); 12% in bereaved Saudi Arabian undergraduate students, (5); 5% in female university students in the United Arab Emirates, (6)]. This discrepancy might be explained by the fact that our sample was treatment-seeking, meaning that the participants were already aware of their mental health problems and thus actively seeking help, albeit not specifically for PGD.

### PGD and its associated characteristics

When linear regression analysis was applied, the following eight variables were significantly associated with higher PGD symptom severity: younger age, female gender, a higher number of experienced or witnessed traumatic event types, having lost a close as opposed to distant family member, a higher number of close lost persons, older age at the time of the loss, a higher subjective retrospectively reported impact of the loss in daily life during the first year, and less perceived social support. When logistic regression analysis was applied, similar, albeit fewer variables significantly increased the likelihood of meeting PG-13 PGD criteria: female gender, having lost a close as opposed to distant family member, a higher number of close lost persons, a higher subjective retrospectively reported impact of the loss in daily life during the first year, and less perceived social support. Thus, five associated factors were found in both regression analyses, and the linear regression yielded three additional significant factors for PGD symptom severity.

The significant associated factors found in this study are consistent with the literature. At this point, we will focus particularly on discussing the five common factors found in both regression analyses. First, as described in the literature (8, 10), female gender was also a factor associated with PGD in the present sample. As with other mental disorders, women seem to have a higher likelihood of developing PGD after bereavement. Second, with regard to the relationship with the deceased, the results are also in line with the literature (1, 2, 8), by demonstrating that losing close family members (i.e., partner, child, parents, and siblings) is associated with a higher risk of developing PGD as compared to losing a distant relative. In our sample, the category of close family members mainly referred to parents. Third, the associated factor of a higher number of losses found in both regressions is consistent with the results of the meta-analysis on violent losses by Heeke et al. (8). This finding is also comparable to previous studies on PTSD, which reported an association between a higher number of traumatic events and greater PTSD symptoms (37). Potentially, this suggests that resilience decreases with an increasing number of losses. Fourth, the retrospectively perceived impairment in the first year after the loss emerged as a factor associated with PGD in our analyses. On average, the loss in our sample occurred 6 years earlier, suggesting that individuals with a current PGD diagnosis according to PG-13 diagnostic criteria may have already perceived psychological problems in the first year of the loss, which had subsequently become chronic. However, these results have to be interpreted with caution, as there may have been a retrospective bias. Nevertheless, a recent longitudinal study likewise demonstrated that people who already showed a high level of grief-associated symptoms in the first year after a loss on average suffered from more severe symptoms and functional impairment 3 years later (38). The final associated factor found in both analyses was the individual perceived social support. Previous studies revealed inconsistent findings with respect to social support. Smith et al. (39) suggested that a sense of social disconnection may act as a barrier to asking for and receiving social support and that this barrier, and thus the ability to feel socially included, may affect the quantity of perceived social support. In addition, it is suggested that a lack of social support might be a precursor to and a result of prolonged grief (40). Due to the large amount of time since the loss in our study, it is likely that the perceived social support is a result of the grief rather than a factor associated with PGD. However, a strong negative relation between social support and PGD symptoms was also shown in another study with an Arabic-speaking population (5).

Three additional factors associated with PGD were only found in the linear regression analysis and will therefore be discussed only briefly. Younger age at the time of assessment, but older age at the time of loss, was associated with increased PGD scores in our study. At first glance, this seems to be in contrast to previous study findings. In meta-analyses (1, 2, 8), age was generally not associated with PGD severity, or only associated as a trend. However, this relationship was consistently positive, indicating that older age might be a factor associated with PGD. One explanation for this different association might be that our sample was rather young and the loss was experienced on average at the age of 19 years. Another study revealed that individuals who experience a loss during adolescence or young adulthood show more severe psychological problems, even after psychosocial counseling, than individuals who do not experience loss at this age (41). Lastly, a higher number of traumatic events was associated with increased PGD severity in our analysis, which is consistent with the meta-analysis conducted by Heeke et al. (8). In our sample, participants experienced or witnessed an average of four traumatic events, with a quite large range from 0 to 23 events. This relatively high number of traumatic events might be attributable to the fact that some countries were affected by war or difficult living situations, which might be connected to a higher number of losses. The finding that the number of traumatic events is only an associated factor for symptom severity, and not for the diagnosis of PGD, might be an additional indication that the event of a loss *per se* is decisive for the development of PGD, and not a traumatic event (if it is not a loss) or the number of traumatic events.

### Comparing PGD severity and PGD diagnostic criteria

The logistic regression yielded fewer factors associated with PGD according to PG-13 diagnostic criteria than did the linear regression with PGD symptom severity scores (five factors were common to both regressions and the linear regression revealed three additional significant factors for PGD symptom severity). As indicated by Singer et al. (10), this difference can be explained by the fact that a continuous score reflects the continuum between normal and dysfunctional grieving, and therefore also a range of associated factors within this continuum, rather than the identification of clinical samples and their valid associated factors. In our sample, however, more overlapping associated factors were found in the two regression analyses than in the study by Singer et al. (10), possibly due to our focus on treatment-seeking individuals and, associated with this, more individuals in our sample meeting the PGD criteria.

### Limitations

The following limitations of our study should be considered. First of all, the generalizability of the results to all Arabic-speaking populations is limited. Recruitment was not conducted by random sampling; rather, individuals interested in treatment for depression and/or PTSD symptoms participated in the screening process. Despite the large sample size, further biases such as a selection bias (i.e., the study appealed to only a particular subgroup of people) cannot be ruled out. Nevertheless, although the sample is not representative of the general population, it does reflect typical samples from internet-based surveys and therapies [e.g., (42)]. Second, the assessment of PGD using the self-administered PG-13 differs from other measures and more profound measurements such as clinical interviews to confirm the diagnosis. In addition, PG-13 items only resemble, but are not identical to ICD-11 PGD and the person's religious and cultural context, which is included in the ICD-11 criteria, cannot be considered in this study and represents a limitation. A third limitation concerns the results regarding professional support structures. The lack of significance for this factor does not necessarily mean that there is no association in general. In this study, we made a first attempt to operationalize a mapping of this variable through external mental health workforce data. In the absence of consistent data from one source for all countries, it was sometimes necessary to draw on different sources and different years. Hence, our analysis comprised only an approximation of these data, and future studies should ideally incorporate a more consistent operationalization. For the time being, however, no such data are available. Fourth, the cross-sectional design of the present study does not allow for conclusions about causal relationships between the variables, but only indicates a first estimate of a probable prevalence rate and associated factors in Arabic-speaking populations. Finally, we did not include possible comorbid disorders in the analysis in this study. However, it has been shown in the literature that a co-occurrence with depressive or anxiety symptoms as well as post-traumatic-stress symptoms exists (43). Nevertheless, we assume that the associated factors found are related to PGD and not to general stress after a loss or to post-traumatic stress symptoms in the current study. On the one hand, the sample in this study experienced a loss a long time ago and still shows moderate PGD symptom severity after such a long time. On the other hand, the sample has primarily experienced a non-violent loss of a close relative or grandparent and did not report this event as the most traumatic experience, as well as having experienced few traumatic experiences in general. Nevertheless, we cannot exclude the possibility that other comorbid problems, such as depressive or anxiety symptoms, have an influence on these specific factors associated with PGD which are consistent with the current literature.

### Future directions and conclusion

The present study examined PGD for the first time in a larger sample from different Arabic-speaking populations, thus providing initial insights into this population in the context of this recently introduced disorder. The aforementioned limitations also provide directions for future research to substantiate or verify the present findings. It has been shown that it is important for longitudinal studies to examine a heterogeneous group (e.g., age, gender, and type of loss) and to focus on both the diagnostic criteria and the symptom severity. When looking at continuous measures and symptom severity it could be beneficial to analyze pathways of severity and determine if factors associated with PGD differ, respectively, e.g., by splitting up samples on the basis of the time after the loss and separately inspecting the determining factors.

Even though 40% of the variance in the present study was already explained by the examined associated factors, it is crucial to ascertain whether there may be additional influences, for example feelings of guilt (44) or unresolved conflicts (45).

An essential aspect of this study is the substantial proportion of participants who met the criteria for PG-13 PGD (as an approximation of ICD-11 diagnostic criteria), revealing that therapeutic services are indispensable in this population. As PGD is a distinct disorder in the ICD-11 (3), it requires a specific therapeutic approach. However, the existing professional support structures for many Arabic-speaking populations are not sufficient to provide effective and timely therapies. Non-professional social support, while having proven to be helpful, is not sufficient or adequate to recover from an existing and possibly chronic mental health disorder. The finding that young women in our study were at particularly high risk of developing PGD, and are already a particularly vulnerable group in many countries, underlines the necessity to ensure that adequate treatment options are made available in order to promote personal development, participation in society, and the empowerment of women.

## Data availability statement

The detailed sociodemographic information of the dataset does not fully protect the anonymity of the respondents. For this reason, the entire dataset cannot be made publicly available. However, excerpts of the data on a higher aggregation level can be provided upon justified request to the corresponding author.

## Ethics statement

The studies involving human participants were reviewed and approved by Freie Universität Berlin. The patients/participants provided their written informed consent to participate in this study.

## Author contributions

BW, CK, and MB designed the overall study project. FS and MV carried out the statistical analyses. FS, MV, NS, and MB drafted the manuscript. FS, MV, MB, NS, BW, and CK carefully revised the manuscript. All authors contributed to the article and approved the submitted version.

## Funding

This project was funded by Misereor e.V. (Grant Number 800-900-1030). Misereor had no role in study design, data collection, analysis, and interpretation as well as the decision to submit the article.

## Conflict of interest

The authors declare that the research was conducted in the absence of any commercial or financial relationships that could be construed as a potential conflict of interest.

## Publisher's note

All claims expressed in this article are solely those of the authors and do not necessarily represent those of their affiliated organizations, or those of the publisher, the editors and the reviewers. Any product that may be evaluated in this article, or claim that may be made by its manufacturer, is not guaranteed or endorsed by the publisher.
