# Supplementary material for: Prolonged grief disorder in Arabic-speaking treatment-seeking populations: Relationship with socio-demographic aspects, loss- and trauma-related characteristics, and mental health support
Source: Front Psychiatry. 2022 Sep 15;13:933848. doi: 10.3389/fpsyt.2022.933848 (PMC9520198; doi:10.3389/fpsyt.2022.933848)
Supplement: Supplementary file 1 [file Data_Sheet_1.PDF]

Supplementary Material

Table 1

List of countries of residence and their mental health workforce capacity

Country	Data source	Year	psychiatrists per 100,000 inhabitants	psychologists per 100,000 inhabitants
Afghanistan	WHO	2016	0.231	0.296
Algeria	WHO	2014	2.290	3.430
Austria	OECD	2020	18.000	118.00
Bahrain	WHO	2017	5.467	1.239
Belgium	WHO	2013	20.057	10.462
Bulgaria	WHO	2016	7.203	1.867
Canada	WHO	2017	14.679	48.740
Denmark	WHO	2014	9.570	12.780
Egypt	WHO	2016	1.600	0.256
France	WHO	2017	20.907	48.704
Germany	WHO	2015	13.202	49.555
Indonesia	WHO	2016	0.310	0.175
Iraq	WHO	2017	0.343	0.111
Ireland	OECD	2020	19.00	–
Israel	WHO	2016	9.870	88.089
Italy	WHO	2015	5.978	3.798
Jordan	WHO	2016	1.125	1.266
Kenya	WHO	2016	0.184	–
Kuwait	WHO	2014	3.330	2.100
Lebanon	WHO	2015	1.213	3.298
Libya	Okasha et al. (2012)	2007	0.200	–
Malaysia	WHO	2016	1.048	1.029
Mexico	WHO	2016	0.207	3.459
Morocco	WHO	2016	0.839	0.575
Netherlands	WHO	2015	20.870	123.464
Oman	WHO	2015	1.738	0.786
Palestine	WHO	2006	0.870	0.980

Poland	WHO	2016	24.176	16.346
Portugal	WHO	2014	4.490	2.240
Qatar	WHO	2016	2.712	1.413
Republic of Korea	WHO	2016	5.793	1.591
Russian Federation	WHO	2015	8.479	4.638
Saudi Arabia	WHO	2016	1.321	2.034
Somalia	WHO	2017	–	0.020
Sudan	WHO	2015	0.080	–
Sweden	WHO	2016	20.863	–
Syria	WHO	2016	0.368	1.068
Tunisia	WHO	2017	–	0.009
Turkey	WHO	2016	1.637	2.537
Ukraine	WHO	2016	6.930	–
United Arab Emirates	WHO	2016	1.649	0.765
United Kingdom	WHO	2014	211.720	12.830
United States of America	WHO	2016	10.542	29.864
Vietnam	WHO	2014	0.910	0.090
Yemen	WHO	2016	0.201	0.409

Table 2

Full list of countries of origin and residence

	Country of origin *n* (%)	Country of residence *n* (%)
Egypt	340 (32.4)	339 (32.3)
Saudi Arabia	203 (19.3)	194 (18.5)
Jordan	75 (7.1)	78 (7.4)
Syria	65 (6.2)	7 (0.7)
Morocco	61 (5.8)	59 (5.6)
Iraq	45 (4.3)	35 (3.3)
Algeria	39 (3.7)	37 (3.5)
United Arab Emirates	39 (3.7)	39 (3.7)
Kuwait	29 (2.8)	25 (2.4)
Tunisia	21 (2.0)	20 (1.9)
Yemen	19 (1.9)	4 (0.4)
Oman	18 (1.7)	19 (1.8)
Bahrain	17 (1.6)	17 (1.6)
Sudan	15 (1.4)	15 (1.4)
Libya	14 (1.3)	9 (0.9)
Palestine	13 (1.2)	15 (1.4)
Lebanon	11 (1.0)	6 (0.6)
Qatar	7 (0.7)	7 (0.7)
Germany	3 (0.3)	41 (3.9)
Israel	3 (0.3)	2 (0.2)
Ethiopia	2 (0.2)	–
Somalia	2 (0.2)	1 (0.1)
United Kingdom	2 (0.2)	5 (0.5)
El Salvador	1 (0.1)	–
France	1 (0.1)	4 (0.4)
Ghana	1 (0.1)	–
Italy	1 (0.1)	3 (0.3)
Madagascar	1 (0.1)	–
Somaliland	1 (0.1)	–

Ukraine	1 (0.1)	3 (0.3)
United States of America	1 (0.1)	8 (0.8)
Turkey	–	29 (2.8)
Canada	–	4 (0.4)
Russian Federation	–	4 (0.4)
Sweden	–	4 (0.4)
Austria	–	4 (0.4)
Denmark	–	2 (0.2)
Afghanistan	–	1 (0.1)
Belgium	–	1 (0.1)
Bulgaria	–	1 (0.1)
Indonesia	–	1 (0.1)
Ireland	–	1 (0.1)
Kenya	–	1 (0.1)
Malaysia	–	1 (0.1)
Mexico	–	1 (0.1)
Netherlands	–	1 (0.1)
Poland	–	1 (0.1)
Portugal	–	1 (0.1)
Republic of Korea	–	1 (0.1)
Vietnam	–	1 (0.1)

Note. $N = 1{,}051$.

Table 3

List of traumatic event types reported by participants

Trauma type	*n* (%)
Sexual contact while under the age of 18 with a person at least 5 years older (e.g., contact with genitals or breasts)	428 (16.3)
Being close to death	389 (14.8)
Poor health without access to medical care	327 (12.5)
Unnatural death of a family member or friend	293 (11.2)
Life-threatening illness	246 (9.4)
Serious accident, fire or explosion (e.g., industrial accident, agricultural accident, car accident, airplane or ship accident)	222 (8.5)
Lack of food or water	211 (8.0)
Sexual assault by a family member or acquaintance (e.g., rape or attempted rape)	206 (7.8)
Violent attack by a family member or acquaintance (e.g., being physically attacked, robbed, shot or threatened with a firearm, stabbed)	185 (7.0)
Sexual assault by stranger (e.g., rape or attempted rape)	185 (7.0)
Violent assault by stranger (e.g., being physically assaulted, robbed, shot at or threatened with a firearm, being stabbed)	164 (6.2)
Serious injury	162 (6.2)
Combat deployment in war or stay in war zone	151 (5.8)
Murder of a family member or friend	132 (5.0)
Murder of a stranger or strangers	124 (4.7)
Natural disaster (e.g., hurricane, tornado, flood disaster, severe earthquake)	112 (4.3)
Forced separation of family members	111 (4.2)
Torture	109 (4.2)
Not having a roof over your head	97 (3.7)
Forced isolation	87 (3.3)
Disappearance or kidnapping	80 (3.0)
Captivity (e.g., prisoner of punishment, prisoner of war, hostage)	71 (2.7)
Serious injury, damage or death caused to someone else by you	55 (2.1)
Brainwashing	54 (2.1)

Note. N = 1,051. Multiple answers possible.
